# Nanosecond pump–probe device for time-resolved serial femtosecond crystallography developed at SACLA

**DOI:** 10.1107/S160057751701030X

**Published:** 2017-08-22

**Authors:** Minoru Kubo, Eriko Nango, Kensuke Tono, Tetsunari Kimura, Shigeki Owada, Changyong Song, Fumitaka Mafuné, Ken Miyajima, Yoshihiro Takeda, Jun-ya Kohno, Naoya Miyauchi, Takanori Nakane, Tomoyuki Tanaka, Takashi Nomura, Jan Davidsson, Rie Tanaka, Michio Murata, Takashi Kameshima, Takaki Hatsui, Yasumasa Joti, Richard Neutze, Makina Yabashi, So Iwata

**Affiliations:** a RIKEN SPring-8 Center, 1-1-1 Kouto, Sayo, Hyogo 679-5148, Japan; b PRESTO, Japan Science and Technology Agency, 4-1-8 Honcho, Kawaguchi, Saitama 332-0012, Japan; c Department of Cell Biology, Graduate School of Medicine, Kyoto University, Yoshidakonoe-cho, Sakyo-ku, Kyoto 606-8501, Japan; d Japan Synchrotron Radiation Research Institute, 1-1-1 Kouto, Sayo, Hyogo 679-5198, Japan; e Department of Chemistry, Graduate School of Science, Kobe University, 1-1 Rokkodai, Nada-ku, Kobe 657-8501, Japan; f Department of Physics, Pohang University of Science and Technology, Pohang 790-784, Korea; g Department of Basic Science, School of Arts and Sciences, The University of Tokyo, Komaba, Meguro, Tokyo 153-8902, Japan; h East Tokyo Laboratory, Genesis Research Institute, Inc., Futamata, Ichikawa, Chiba 272-0001, Japan; i Department of Chemistry, School of Science, Gakushuin University, Mejiro, Toshima, Tokyo, Japan; j Research Center for Advanced Measurement and Characterization, National Institute for Materials Science, Sengen, Tsukuba, Ibaraki 305-0047, Japan; k Department of Biological Sciences, Graduate School of Science, The University of Tokyo, 7-3-1 Hongo, Bunkyo-ku, Tokyo 113-0033, Japan; l Department of Chemistry, Ångström Laboratory, Uppsala University, Uppsala, Sweden; m JST–ERATO, Lipid Active Structure Project, Osaka University, 1-1 Machikaneyama, Toyonaka, Osaka 560-0043, Japan; n Department of Chemistry and Molecular Biology, University of Gothenburg, Box 462, SE-40530 Gothenburg, Sweden

**Keywords:** XFEL, serial femtosecond crystallography, time-resolved X-ray crystallography, pump and probe

## Abstract

X-ray free-electron lasers (XFELs) have opened new opportunities for time-resolved X-ray crystallography. Here a nanosecond optical-pump XFEL-probe device developed for time-resolved serial femtosecond crystallography (TR-SFX) studies of photo-induced reactions in proteins at the SPring-8 Angstrom Compact free-electron LAser (SACLA) is reported. The optical-fiber-based system is a good choice for a quick setup in a limited beam time and allows pump illumination from two directions to achieve high excitation efficiency of protein microcrystals. Two types of injectors are used: one for extruding highly viscous samples such as lipidic cubic phase (LCP) and the other for pulsed liquid droplets. Under standard sample flow conditions from the viscous-sample injector, delay times from nanoseconds to tens of milliseconds are accessible, typical time scales required to study large protein conformational changes. A first demonstration of a TR-SFX experiment on bacteriorhodopsin in bicelle using a setup with a droplet-type injector is also presented.

## Abstract 2

A nanosecond pump–probe device for time-resolved serial femtosecond crystallography has been developed at SACLA.

## 1. Introduction   

With the development of X-ray free-electron lasers (XFELs), X-ray crystallography of proteins entered a new phase. One of the key emerging techniques is serial femtosecond crystallography (SFX), which uses a continuous flow of microcrystals in random orientations to obtain diffraction images on a single-shot basis (Chapman *et al.*, 2011 ▸; Boutet *et al.*, 2012 ▸). Continuous sample delivery in SFX is advantageous in time-resolved (TR) experiments because activated samples are replaced with fresh samples immediately during data collection. Among various TR techniques, the pump–probe method is well established and can easily be combined with SFX to observe photo-induced reactions (Kern *et al.*, 2014 ▸; Kupitz *et al.*, 2014 ▸; Tenboer *et al.*, 2014 ▸; Pande *et al.*, 2016 ▸; Barends *et al.*, 2015 ▸). The femtosecond laser systems installed at the XFEL facilities [LCLS (Minitti *et al.*, 2015 ▸) and SACLA (Togashi *et al.*, 2014 ▸)] are available for researchers to observe photo-induced reactions with femtosecond time resolution. On ultrafast time scales (femtoseconds to picoseconds), protein motions are local and often involve chromophore or amino acid side chains. In contrast, motions on slower time scales (nanoseconds to milliseconds) might involve larger conformational changes in association with protein function. A pump–probe TR-SFX setup using a nanosecond laser is useful for observing such protein movements on a slower time scale. Here we report the development of an optical-fiber-based setup for a nanosecond pump–probe TR-SFX at SACLA. This simple robust system allows quick setup, and pump conditions (wavelength and focal size) are readily adjustable so users can collect as much data as possible in limited XFEL beam time. In this system, pump light illuminates protein microcrystals from two directions, thus enabling higher-efficiency sample excitation. In this paper, we also reveal an application to bacteriorhodopsin (bR) in bicelle using a droplet injector (Mafuné *et al.*, 2016 ▸), a first demonstration of a TR-SFX experiment with a droplet injector.

## 2. Instrumentation   

### 2.1. Overview of the experimental setup   

**Figure 1:**
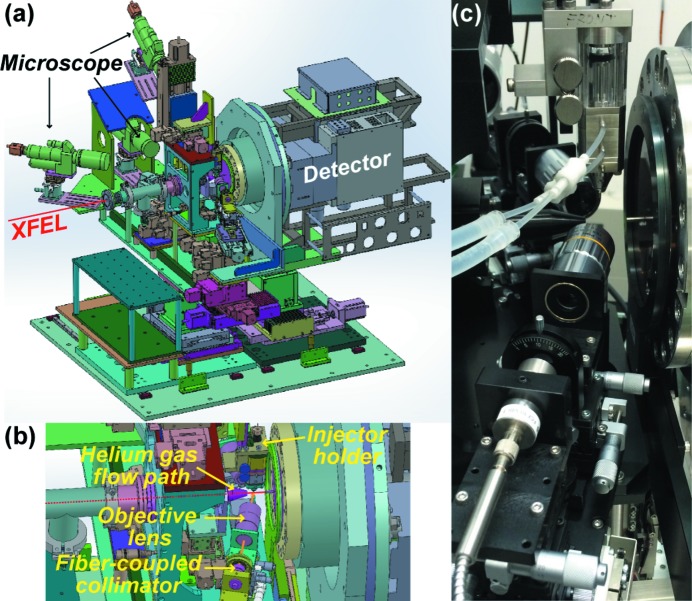
(*a*) Experimental setup for the pump–probe TR-SFX. An enlarged view around the sample is shown in (*b*) and a photograph of the same enlarged view in (*c*).

We previously reported an experimental setup without a pump system for SFX, named DAPHNIS (Diverse Application Platform for Hard X-ray diffractioN In SACLA) (Tono *et al.*, 2015 ▸), which consisted mainly of a helium chamber, an injector holder and a multiport charge-coupled device (MPCCD) detector (Kameshima *et al.*, 2014 ▸). Due to limited space in the chamber, incorporating a pump system into DAPHNIS was not feasible. Therefore, we designed a new experimental setup using a nanosecond laser for TR-SFX, composed of a pump laser system, an injector holder, microscopes and the MPCCD detector (Fig. 1 ▸). Instead of a helium chamber, a helium gas flow path was introduced to reduce background noise from air scattering (Fig. 1*b* ▸). An XFEL beam from the focusing system enters the setup through a beryllium window and passes through the helium gas flow path, intersecting with a sample stream. Samples are delivered to an XFEL focal point using a sample injector mounted on a motorized manipulator. For sample injection, two types of injectors are available: one is for ejecting viscous samples, such as the lipidic cubic phase (LCP) (Weierstall *et al.*, 2014 ▸), and the other is for pulsed liquid droplets (Mafuné *et al.*, 2016 ▸). The viscous-sample injector (Shimazu *et al.*, 2017 ▸) was developed on the LCP injector concept reported by Weierstall *et al.* (2014 ▸), and this setup was utilized in the TR-SFX study of bR in LCP (Nango *et al.*, 2016 ▸). Recently, structural changes of photosystem II (Suga *et al.*, 2017 ▸) were also reported, using the same setup with grease as a carrier medium (Sugahara *et al.*, 2015 ▸). In these studies, sample flow speed was set to 5.3 mm s^−1^ (photosystem II) or 9.4 mm s^−1^ (bR) to avoid light contamination, whereas samples can be injected at slower flow speed (∼1 mm s^−1^) in an SFX experiment without a pump laser. Since microcrystals travel relatively fast during the delay time after photo-excitation, the longest delay time is currently limited to approximately 20 ms. The shortest delay time is 7 ns as described in the following section.

Three microscopes are used for sample flow observation or for alignment of the injector position into the pump and XFEL interaction volume using *XYZ* stages controllable from the outside of the experimental hutch. To protect the detector from the intense primary XFEL beam, a beam stopper is placed on the Kapton window in front of the detector. The Kapton window and a beryllium window of the detector work effectively as filters for pump light scattering. Further to suppress pump light scattering, components around the sample are black. The distance between the MPCCD sensor and the sample is adjustable from 50 to 100 mm. The setup’s maximum resolution is 1.5 Å at a sensor-to-sample distance of 50 mm and an X-ray wavelength of 1.24 Å due to the sensor-area span.

### 2.2. Pump laser system   

Two types of pump laser systems are available.

(i) An optical parametric oscillator (OPO) provides >1 mJ, 6 ns pulses at a wavelength tunable from 300 to 2000 nm, with a repetition rate up to 30 Hz (NT230, EKSPLA). This system can trigger various photo-induced reactions and is promising for photolysis of caged-compounds that can initiate enzymatic reactions. Note that, although various caged-compounds are commercially available (Ellis-Davies, 2007 ▸), they have to be selected so that the uncaging time scale is short enough compared with the time range of the observed protein dynamics.

(ii) The SHG output of an Nd:YAG laser is also available; this delivers 12 mJ, 5 ns pulses at 532 nm with a repetition rate up to 15 Hz (Minilite-I, Continuum). The same two Nd:YAG lasers have been installed, so two-flash excitation is available at 532 nm (Suga *et al.*, 2017 ▸).

Using a 50:50 beamsplitter, the pump beam is divided into two arms, each fed into an optical fiber with a core diameter of 50–400 µm [numerical aperture (NA) 0.22] (Optran UV/WF with high-power SMA, CeramOptec). Optical fibers deliver pump beams near the sample (Fig. 1*b* ▸). Each output beam from a fiber is collimated with a diameter of 10 mm using a fiber-coupled collimator (NA 0.175) (FC10-VIS-T or FC10S-UV1-FC, Micro Laser Systems) and focused on a sample stream using an objective lens (M Plan Apo 10× with NA 0.28, MITSUTOYO or LMU-5X-NUV/-UVB with NA 0.13, THORLABS). Note that optical fibers facilitate the pump beams’ alignment procedure. Because the pump beam paths from the laser to fiber inputs and from fiber outputs to objective lenses are normally fixed, the only thing to perform for pump-beam transportation is fiber attachment. On the other hand, if one used a femtosecond laser in the setup, irradiating enough intense laser pulses to excite samples would be difficult due to the fiber’s damage threshold. Furthermore, second- or higher-order dispersion is liable to extend pulse duration. The two pump beams illuminate two sides of the crystal in a nearly counter-propagating geometry (∼80° off the X-ray beam) to enhance excitation efficiency. The off-axis angle should be greater than 75° to avoid pump beams directly entering the MPCCD.

**Figure 2:**
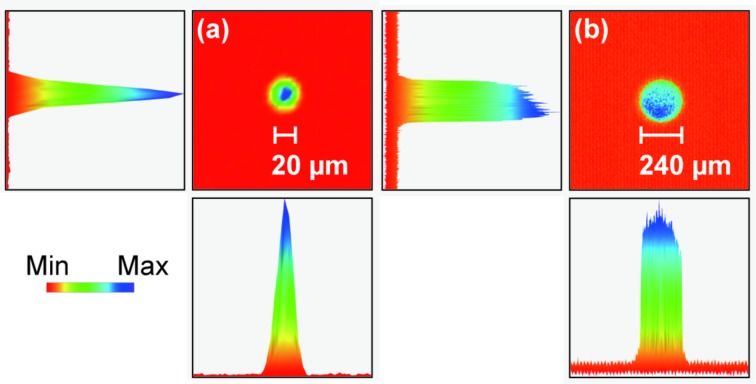
Spatial profile of the 532 nm pump beam using a fiber with a core size of (*a*) 50 µm and (*b*) 400 µm. The beam profile was estimated by a CCD beam profiler (SP620, Ophir-Spiricon).

The pump’s focal size depends on the optics’ combination, but is controllable by fiber core size (50–400 µm). The minimum focal size is 20 µm (FWHM) using a 50 µm core size fiber, while the focal size is expandable to 240 µm (top-hat) using a 400 µm core size fiber (Fig. 2 ▸). A focal size of 40 µm (FWHM) using a 100 µm core size fiber fits a 75 µm-diameter sample stream, and this is a typical setup for TR-SFX using the viscous-sample injector at SACLA (Shimazu *et al.*, 2017 ▸).

The pump laser is synchronized with the XFEL *via* a delay generator (DG645, Stanford Research Systems), triggered by the TTL signal provided to users at 15 ms prior to each XFEL pulse. The delay time between pump and XFEL pulses is adjusted with the delay generator, which enables users’ easy operation. Because the timing jitter is <1 ns, the time resolution is <7 ns. Typically, the pump laser’s repetition rate is set to half of that of the XFEL by the same delay generator in order to collect the alternating ‘light-on’ and ‘light-off’ diffraction data, the latter of which serve as a dark dataset. To discriminate ‘light-on’ or ‘light-off’ data, a portion of the pump beam (5%) is picked off with a beam sampler, and its voltage signal detected by a photodiode tags a diffraction image with ‘light-on’. The photodiode readout is stored in the SACLA data acquisition system’s database, along with other shot-by-shot metadata such as wavelength and beam intensity. The data processing pipeline (Nakane *et al.*, 2016*a* ▸) retrieves data through the SACLA API (Joti *et al.*, 2015 ▸) and then sorts hit images into ‘light-on’ and ‘light-off’ datasets.

The pump beam can be aligned spatially with the XFEL beam using *XYZ* stages controllable from outside of the experimental hutch. First, the pump beam center is aligned to overlap with the XFEL and then is raised using a *Z* stage that depends on a delay time, to illuminate a sample flow’s upper stream. Note here that, when the pump beam center is positioned in the upper stream relative to the XFEL spot, we usually set the pump beam area’s size large enough to overlap spatially with the XFEL spot. (For example, in the case of a sample flow rate of ∼5 mm s^−1^, crystals move ∼100 µm in 20 ms. For measurement with a 20 ms delay time, the pump beam center is aligned and positioned ∼100 µm upstream from the XFEL spot, while the pump beam size is set to greater than 200 µm diameter, so the area covers the XFEL spot.) Such a pump–XFEL spatial overlap contributes to the integrity of ‘light-on’ data, which should be derived from pump-illuminated crystals even if the sample flow rate fluctuates.

## 3. Application   

We performed a TR-SFX experiment on the membrane protein bR, a light-driven proton pump, using the pump–probe TR-SFX device at the EH4c experimental station of SACLA BL3 (Ishikawa *et al.*, 2012 ▸; Tono *et al.*, 2013 ▸). In this experiment, bR crystals in bicelle were loaded with a liquid carrier to the droplet injector, which is synchronized to XFEL pulses (Mafuné *et al.*, 2016 ▸). We crystallized bR in bicelle, prepared by mixing DMPC/CHAPSO with a precipitant solution composed of 3.2 *M* NaH_2_PO_4_, 3.5% tri­ethyl­ene glycol and 180 m*M* 1,6-hexandiol (Faham *et al.*, 2005 ▸; Nakane *et al.*, 2016*b* ▸). bR crystals including the precipitant mixture were ejected from the droplet injector equipped with a 80 µm nozzle. The amplitude and duration of the electric pulse for driving the droplet injector were set to 90 V and 90 µs, respectively. Data were collected using 7.6 keV X-ray pulses with a duration of <10 fs at 30 Hz. The pump laser (Minilite-I, Continuum), with a 532 nm wavelength, was operated at 15 Hz to provide ‘light-on’ and ‘light-off’ interleaved images. The pump focal diameter was 160 µm (FWHM), and its intensity was 8 µJ at the sample point. The delay time between pump and XFEL pulses was set to 1 µs. The droplet’s flow speed was ∼14 m s^−1^. While the illuminated droplet traveled 14 µm downward during a delay time of 1 µs, the droplet was so large (80 µm in diameter) that it could interact with the XFEL beam. Therefore, under the present experimental conditions, the longest delay time is limited to approximately 2 µs.

**Figure 3:**
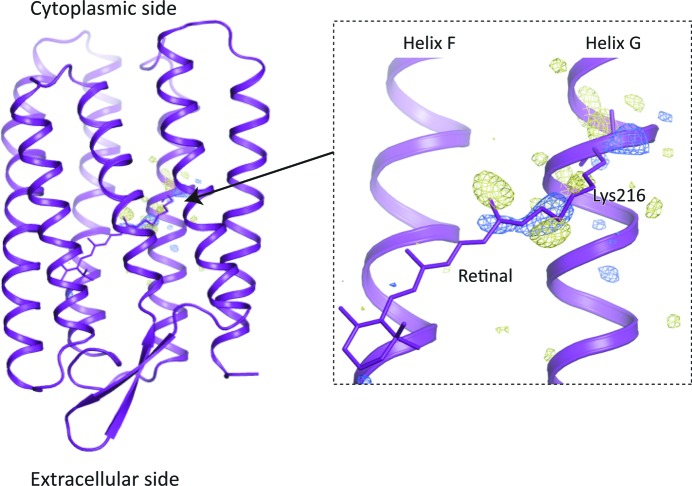
*F*(light) − *F*(dark) difference electron density for 1 µs time delay data. Blue represents a positive difference density (contoured at +3.5σ) and yellow a negative difference density (contoured at −3.5σ).

bR crystals diffracted to 2.2 Å. A total of 371648 diffraction patterns were collected, of which 20418 (20890) were identified as hit images in the light (dark) condition, with more than 20 potential Bragg peaks, by using the data processing pipeline (Nakane *et al.*, 2016*a* ▸) based on *Cheetah* (Barty *et al.*, 2014 ▸). By further processing in *CrystFEL* version 0.6.1 (White *et al.*, 2012 ▸), 16186 (16548) indexed images were obtained as light (dark) data (Table S1 of the supporting information). A difference Fourier map was calculated from the light/dark datasets using the CCP4 suite (Winn *et al.*, 2011 ▸) and showed structural changes on the periphery of the retinal that underwent isomerization in response to light (Fig. 3 ▸). Positive and negative difference densities appeared on the retinal and around Lys216 of helix G; this indicates structural changes at a relatively early stage in the bR photocycle. Whereas, to maintain a smooth sample flow, monoolein and liquid paraffin were added to the bR-crystal sample in LCP before measurement in the TR-SFX experiment (Nango *et al.*, 2016 ▸), the droplet injector allows injection of pristine crystals without any additional agents. The combination of the droplet injector and the pump–probe TR-SFX device would be beneficial for observing structural changes on protein crystals sensitive to additives.

## 4. Conclusions   

We have developed a setup for nanosecond pump–probe TR-SFX at SACLA and successfully applied it to bR in bicelle by using a pulsed liquid droplet injector. Nanosecond time resolution is enough for biological applications, particularly when studying proteins’ large functional motions. Moreover, the nanosecond system is simple, robust and easy to operate, allowing users to collect as much data as possible in limited beam time. One of the most powerful ways to expand applications of pump–probe TR-SFX would be to use caged-compounds. The present system, which can provide various pump wavelengths from UV to near IR, is compatible with such applications.

## 5. Related literature   

The following reference is cited in the supporting information for this article: Duisenberg (1992 ▸).

## Supplementary Material

Crystallographic statistics. DOI: 10.1107/S160057751701030X/rv5066sup1.pdf
