# Supplementary material for: Nanosecond pump–probe device for time-resolved serial femtosecond crystallography developed at SACLA
Source: J Synchrotron Radiat. 2017 Aug 22;24(Pt 5):1086–91. doi: 10.1107/S160057751701030X (PMC5580792; doi:10.1107/S160057751701030X)
Supplement: Supplementary file 1 [file s-24-01086-sup1.pdf]

JOURNAL OF SYNCHROTRON RADIATION

Volume 24 (2017)

Supporting information for article:

Nanosecond pump–probe device for time-resolved serial femtosecond crystallography developed at SACLA

Minoru Kubo, Eriko Nango, Kensuke Tono, Tetsunari Kimura, Shigeki Owada, Changyong Song, Fumitaka Mafuné, Ken Miyajima, Yoshihiro Takeda, Jun-ya Kohno, Naoya Miyauchi, Takanori Nakane, Tomoyuki Tanaka, Takashi Nomura, Jan Davidsson, Rie Tanaka, Michio Murata, Takashi Kameshima, Takaki Hatsui, Yasumasa Joti, Richard Neutze, Makina Yabashi and So Iwata

Table S1 Crystallographic statistics. Values in parentheses are for the outermost shell.

**Data collection**	Light data	Dark data
Wavelength (Å)	1.63	
Space group	$C222_1$	
Unit-cell parameter		
a (Å)	46.2	
b (Å)	103.0	
c (Å)	128.4	
Number of collected images	371,648	
Number of hits	20,418	20,890
Number of indexed patterns*	16,186	16,548
Indexing rate (%)§	4.4	4.5
Number of merged images#	9,460	10,194
Number of total reflections	3,381,106	3,680,032
Number of unique reflections	16,276	16,276
Resolution range (Å)	38.9–2.2	
Completeness (%)	100 (100)	100 (100)
R_{split} (%)†	8.96 (81.7)	8.74 (65.0)
$CC_{1/2}$	99.2 (55.1)	98.8 (60.8)
$<I/\sigma(I)>$	7.45 (1.49)	7.90 (1.82)

*DirAx (Duisenberg, A., 1992) was used for indexing.

§Percentage of images that were indexed.

#Intensities from images diffracted to better than 3.5 Å were merged by Monte Carlo integration with frame-wise scaling as implemented in the process_hkl command in the CrystFEL suite (White *et al.*, 2012).

$$^{\dagger}R_{split} = 1/\sqrt{2}\frac{\sum_{hkl}|I_{even}-I_{odd}|}{1/2\ \sum_{hkl}|I_{even}+I_{odd}|}$$

Supplementary references

Duisenberg, A. (1992). *J. Appl. Cryst.* 25, 92-96.

White, T. a., Kirian, R. a., Martin, A. V., Aquila, A., Nass, K., Barty, A. & Chapman, H. N. (2012). *J. Appl. Cryst.* 45, 335-341.
